# Severe Imported *Plasmodium falciparum* Malaria, France, 1996–2003

**DOI:** 10.3201/eid1705.101527

**Published:** 2011-05

**Authors:** Elise Seringe, Marc Thellier, Arnaud Fontanet, Fabrice Legros, Olivier Bouchaud, Thierry Ancelle, Eric Kendjo, Sandrine Houze, Jacques Le Bras, Martin Danis, Rémy Durand, for the French National Reference Center for Imported Malaria Study Group

**Affiliations:** Author affiliations: University Pierre et Marie Curie, Paris, France (E. Seringe, M. Thellier, M. Danis);; Groupe Hospitalier Pitié-Salpĕtrière, Paris (E. Seringe, M. Thellier, M. Danis);; Centre National de Référence du Paludisme, Paris (M. Thellier, F. Legros, E. Kendjo, S. Houze, J. Le Bras, M. Danis, R. Durand);; Institut Pasteur, Paris (E. Seringe, A. Fontanet);; Conservatoire National des Arts et Métiers, Paris (A. Fontanet);; Hôpital Avicenne, Bobigny, France (O. Bouchaud, R. Durand);; Hôpital Cochin, Paris (T. Ancelle)

**Keywords:** Imported malaria, Plasmodium falciparum, severe malaria, parasite, vector-borne infections, malaria, France, research

## MEDSCAPE CME

Medscape, LLC is pleased to provide online continuing medical education (CME) for this journal article, allowing clinicians the opportunity to earn CME credit.

This activity has been planned and implemented in accordance with the Essential Areas and policies of the Accreditation Council for Continuing Medical Education through the joint sponsorship of Medscape, LLC and Emerging Infectious Diseases. Medscape, LLC is accredited by the ACCME to provide continuing medical education for physicians.

Medscape, LLC designates this Journal-based CME activity for a maximum of 1 *AMA PRA Category 1 Credit(s)*^TM^. Physicians should claim only the credit commensurate with the extent of their participation in the activity.

All other clinicians completing this activity will be issued a certificate of participation. To participate in this journal CME activity: (1) review the learning objectives and author disclosures; (2) study the education content; (3) take the post-test and/or complete the evaluation at www.medscape.org/journal/eid; (4) view/print certificate.

**Release date: April 22, 2011; Expiration date: April 22, 2012**

## Learning Objectives

Upon completion of this activity, participants will be able to:

- Describe the diagnosis and prognosis of malaria among travelers
- Distinguish characteristics of returning travelers with malaria in the current study
- Analyze variables associated with a higher risk for severe malaria among returning travelers

## MEDSCAPE CME EDITOR

**Beverly Merritt, **Technical Writer/Editor, *Emerging Infectious Diseases. Disclosure: Beverly Merritt has disclosed no relevant financial relationships.*

## MEDSCAPE CME AUTHOR

**Charles P. Vega, MD**, Associate Professor; Residency Director, Department of Family Medicine, University of California, Irvine. *Disclosure: Charles P. Vega, MD, has disclosed no relevant financial relationships.*

## AUTHORS

*Disclosures:* ***Elise Seringe, MD; Marc Thellier, MD; Arnaud Fontanet, MD; Fabrice Legros, MD; Olivier Bouchaud, PhD; Thierry Ancelle, MD; Eric Kendjo; Sandrine Houze, MD; Martin Danis, MD;*** *and* ***Rémy Durand, MD*** *, have disclosed no relevant financial relationships.* ***Jacques Le Bras, PhD,*** *has disclosed the following relevant financial relationships: received grants for clinical research from GlaxoSmithKline; received grant from GlaxoSmithKline on application of AFSSAPS phase 4 requirement on Malarone.*
