# Supplementary material for: Severe Imported Plasmodium falciparum Malaria, France, 1996–2003
Source: Emerg Infect Dis. 2011 May;17(5):807–13. doi: 10.3201/eid1705.101527 (PMC3321778; doi:10.3201/eid1705.101527)
Supplement: Technical Appendix — Members of the National Reference Center Study Group for Imported Malaria in Metropolitan France. [file 10-1527-Techapp_3p.pdf]

Article DOI: 10.3201/eid1705.101527

Severe Imported *Plasmodium falciparum* Malaria, France, 1996–2003

Technical Appendix

Members of the National Reference Center Study Group for Imported Malaria in Metropolitan France: C. Garabedian (Centre Hospitalier, Aix en Provence); M. Greze (Centre Hospitalier Général d'Albi); B. Tourrand (Centre Hospitalier, d'Alès); J. Chandenier and G. Nevez (Centre Hospitalier, Universitaire d'Amiens Hôpital Sud); L. De Gentile and D. Chabasse (Centre Hospitalier Universitaire d'Angers); C. Garandeau (Centre Hospitalier d'Angoulême); J.P. Bru and M. Bensalem (Centre Hospitalier de la Région d'Annecy); M. Roumier (Centre Hospitalier Général); Joseph Imbert d'Arles and A. Chapelle (Centre Hospitalier d'Aubenas); H. Broutier and D. Lusina (Centre Hospitalier Intercommunal); Robert Ballanger d'Aulnay-sous-bois, A. Azzedine, and G. Lepeu (Centre Hospitalier d'Avignon); L. Ducout (Centre Hospitalier de Bayonne); J. Heurtet (Centre Hospitalier de Beauvais); G. Cellier-Julienne and P. Dussert (Centre Hospitalier de Belfort); R. Piarroux, L. Million, and M. Briand (Centre Hospitalier Universitaire de Besançon); R.M. Julien (Centre Hospitalier de Béziers); L. Estepa (Centre Hospitalier de Blois); O. Lortholary and C. Bouges-Michel (Hôpital Avicenne de Bobigny); A. Collignon, I. Poilane, and D. Bemba (Hôpital Jean Verdier de Bondy); P. Millet, M.C. Receveur, and D. Malvy (Centre Hospitalier Universitaire de Bordeaux); J. Dunand and T. Hanslik (Hôpital Ambroise Paré de Boulogne-Billancourt); G. Nevez, E. Moalic, and D. Quinio (Centre Hospitalier Universitaire de Brest); O. Bandin (Hôpital Sainte-Camille de Bry-sur-Marne); C. Duhamel (Centre Hospitalier Universitaire de Caen); C. Grasmick (Centre Hospitalier Général); Jean Rougier de Cahors, O. Rogeaux, and D. Raffenot (Centre Hospitalier Général de Chambery); J.M. Galempoix and C. Penalba (Hôpital de Charleville-Mézières); D. Pons, M. Cambon, and J. Beytout (Centre Hospitalier Universitaire de Clermont-Ferrand); I. Mazurier (Centre Hospitalier Louis Pasteur de Colmar); C. Laurent, G. Galeazzi, and E. Mortier (Hôpital Louis Mourier de Colombes); C. Malbrunot (Centre Hospitalier de Corbeil Essonne); C. van Batten (Centre Hospitalier Laennec de Creil); M. Deniau and S. Bretagne (Centre Hospitalier Universitaire Henri Mondor); E. Estrangin and N. Fauchet (Centre Hospitalier Intercommunal de

Créteil); B. Cuisenier and A. Bonnin (Centre Hospitalier Universitaire Le Bocage); C. Finot (Centre Hospitalier de Dreux); A. Verhaeghe and E. Buffet (Centre Hospitalier Intercommunal d'Elbeuf); A. Gardrat (Hôpital d'Evreux); P. Cormier (Centre Hospitalier Louise Michel d'Evry); J.B. Poux (Centre Hospitalier du Val d'Ariège); C. Braidy (Centre Hospitalier de Fontainebleau); C. Perronne, E. de Truchis, and F. Ader (Hôpital Raymond-Poincaré [Garches]); D. Troisvallets and E. Vandemeulebroucke (Centre Hospitalier de Gonesse); D. Maubon and H. Pelloux (Centre Hospitalier Universitaire de Grenoble); Y. Costa (Centre Hospitalier Général de Lagny sur Marne); D. Jan (Centre Hospitalier Général de Laval); F. Kasawat and J.P. Hurst (Hôpital Jacques Monod); E. Casalino, P. Bourée, and F. Botterel (Hôpital Bicêtre); E. Boyer (Centre Hospitalier Général du Mans); D. Camus, E. Dutoit, and L. Delahes (Centre Hospitalier Régional Universitaire de Lille); M.L. Darde and D. Azjenberg (Centre Hospitalier Universitaire de Limoge); P. Barbut (Centre Hospitalier de Longjumeau); F. Peyron and C. Ramade (Hôpital de la Croix Rousse de Lyon); S. Picot and F. de Monbrison (Université Claude Bernard de Lyon); M.L. Bigel and F. Moussel (Hôpital François Quesnay de Mantes-la-Jolie); H. Dumon and B. Faugère (Hôpital de la Timone de Marseille); F. Simon (Hôpital Laveran de Marseille); P. Parola, J. Delmont, and P. Minodier (Hôpital Nord de Marseille); A. Vincenot and A.E. Andre-Kerneis (Centre Hospitalier Général de Meaux); J.M. Martelli and M. Le Neveux (Centre Hospitalier Intercommunal de Meulan-les-Mureaux); D. Landreau (Centre Hospitalier de Montauban); C. Febvre (Centre Hospitalier Général André Boulloche de Montbeliard); D. Basset and J.P. Dedet (Centre Hospitalier Universitaire de Montpellier), A. Trevoux and J. Delarbre (Hôpital Moenchsberg de Mulhouse); M. Machouart and J.C. Burdin (Centre Hospitalier Universitaire de Nancy); F. Gay-Andrieu, F. Raffiand, and M. Miegeville (Centre Hospitalier Universitaire de Nantes); P. Delaunay, P. Marty,and P. Dellamonica (Centre Hospitalier Universitaire de Nice); L. Lachaud (Centre Hospitalier Universitaire de Nimes); B. Buret (Centre Hospitalier de Niort); D. Poisson (Centre Hospitalier Régional d'Orléans); C. Sarfati (Hôpital Saint-Louis de Paris); M. Cornet (Hôpital Hôtel-Dieu de Paris); P. Ralaimazava, E. d'Ortenzio, L. Vaslin, S. Lariven, S. Matheron, and F. Bruneel (Hôpital Bichat-Claude Bernard de Paris); O. Fenneteau and A. Faye (Hôpital Robert Debré de Paris), H. Yera, and J. Dupouy-Camet (Hôpital Cochin de Paris), M.E. Bougnoux, S. Challier, and B. Dupont (Hôpital Necker de Paris); A. Faussart, F. Gay, E. Caumes, and P. Hausfater (Hôpital Pitié-Salpêtrière de Paris); G. Belkadi, J. L. Poirot, and P. Roux (Hôpital Saint-Antoine de Paris); M. Develoux (Hôpital Tenon de Paris);

H. Lapillonne, B. Quinet, and D. Pop-Jora (Hôpital Trousseau de Paris); L. Gutmann, E. Dannaoui, and V. Lavarde (Hôpital Européen Georges Pompidou à Paris); P. Buffet and A.S. LeGuern (Institut Pasteur à Paris); A. Martin (Centre Hospitalier de Périgueux); M.H. Rodier, C. Kauffmann-Lacroix, G. Lemoal, and F. Roblot (Centre Hospitalier Universitaire de Poitiers); A. Sar and M. Thibault (Hôpital René Dubos de Pontoise); F. Geffroy (Centre Hospitalier Intercommunal de Cornouaille); D. Toubas, C. Chemla, and J. M. Pinon (Hôpital Maison Blanche de Reims); C. Guiguen, C. Michelet, and S. Chevrier (Hôpital Pontchaillou de Rennes); L. Favennec, P. Abboud, and G. Gargala (Centre Hospitalier Régional Universitaire de Rouen); J. Vaucel and E. Duhamel (Centre Hospitalier de Saint-Brieuc); N. Godineau, D. Mechali, and J. Y. Siriez (Hôpital Delafontaine de Saint -Denis); R. Tran Manh Sung and P. Flori (Centre Hospitalier Régional Universitaire de Saint-Etienne); M. Chiron and A. Boisivon (Hôpital de Saint-Germain-en-Laye); J.D. Cavallo, T. Debord, and C. Rapp (Hôpital d'Instruction des Armées Begin de Saint-Mandé); J. Cuziat (Centre Hospitalier de Saint-Nazaire); P. Clergeau (Centre Hospitalier de Sallanches); C. Charrel (Centre Hospitalier de Salon de Provence); A. Barrans (Centre Hospitalier Général de Sète); E. Candolfi, R. Dahan, A. Aboubacar, Y. Hansmann, and D. Christmann (Centre Hospitalier Universitaire de Strasbourg); C. Martinaud and G. Menard (Hôpital d'Instruction des Armées Saint-Anne deToulon); F. Benoit-Vical, B. Marchou, A. Berry, and J.F. Magnaval (Centre Hospitalier, Universitaire Rangueil de Toulouse); P. Patoz (Hôpital Gustave Dron de Tourcoing); T.H. Duong, F. de Closets, and D. Richard-Lenoble (Centre Hospitalier Universitaire de Tours); A. Fur, F. Benaoudia, and J.C. Croix (Centre Hospitalier de Troyes); H. Moindrot, J. Bronner, and M. Vasselon (Centre Hospitalier de Valence); S. Lhopital (Centre Hospitalier Général Saint-Louis de Valence); O. Eloy (Hôpital André Mignot de Versailles); A. Royer (Centre Hospitalier Paul Morel de Vesoul); O. Patey, C. Lapierre, and A. Fisch (Centre Hospitalier Intercommunal de Villeneuve-Saint-Georges); and R. Mazataud (Centre Hospitalier de Vitry le François).
