# Supplementary material for: Severe Imported Plasmodium falciparum Malaria, France, 1996–2003
Source: Emerg Infect Dis. 2011 May;17(5):807–13. doi: 10.3201/eid1705.101527 (PMC3321778; doi:10.3201/eid1705.101527)
Supplement: Related texts

# Research article: Severe Imported *Plasmodium falciparum* Malaria, France, 1996–2003

**DOI:** 10.3201/eid1705.101527

**Authors:** Elise Seringe, Marc Thellier, Arnaud Fontanet, Fabrice Legros, Olivier Bouchaud, Thierry Ancelle, Eric Kendjo, Sandrine Houze, Jacques Le Bras, Martin Danis, Rémy Durand, for the French National Reference Center for Imported Malaria Study Group

**Affiliations:** Author affiliations: University Pierre et Marie Curie, Paris, France (E. Seringe, M. Thellier, M. Danis);; Groupe Hospitalier Pitié-Salpĕtrière, Paris (E. Seringe, M. Thellier, M. Danis);; Centre National de Référence du Paludisme, Paris (M. Thellier, F. Legros, E. Kendjo, S. Houze, J. Le Bras, M. Danis, R. Durand);; Institut Pasteur, Paris (E. Seringe, A. Fontanet);; Conservatoire National des Arts et Métiers, Paris (A. Fontanet);; Hôpital Avicenne, Bobigny, France (O. Bouchaud, R. Durand);; Hôpital Cochin, Paris (T. Ancelle)

## Abstract

Little is known about severe imported *Plasmodium falciparum* malaria in industrialized countries where the disease is not endemic because most studies have been case reports or have included <200 patients. To identify factors independently associated with the severity of *P. falciparum*, we conducted a retrospective study using surveillance data obtained from 21,888 *P. falciparum* patients in France during 1996–2003; 832 were classified as having severe malaria. The global case-fatality rate was 0.4% and the rate of severe malaria was ≈3.8%. Factors independently associated with severe imported *P. falciparum* malaria were older age, European origin, travel to eastern Africa, absence of chemoprophylaxis, initial visit to a general practitioner, time to diagnosis of 4 to 12 days, and diagnosis during the fall–winter season. Pretravel advice should take into account these factors and promote the use of antimalarial chemoprophylaxis for every traveler, with a particular focus on nonimmune travelers and elderly persons.

Each year, a growing number of persons from industrialized countries travel to developing countries. Among these millions of travelers, 20% to 70% report some illness associated with their travel and ≈3% report fever (*1*). Malaria appears to be the most common cause of fever in returned travelers. Because of its potential severity, *Plasmodium falciparum* infection must be considered in all febrile persons who return from an area where malaria is endemic. Depending on the country of importation and the year, ≈2%–16% of *P. falciparum* imported malaria infections are severe cases according to the World Health Organization (WHO) definition, and ≈10% of severe cases end with the death of the patient despite appropriate antimalarial treatment (*2*).

Globally, epidemiologic and pathophysiologic research studies on malaria are mainly based on disease in children <5 years of age in areas where malaria is endemic. Little is known about severe imported malaria, which primarily affects nonimmune adults. Most previous studies that have focused on severe imported malaria have been case reports or have included <200 patients (*3**–**12*). Recently, we published an analysis of risk factors associated with death in a series of 96 malaria-related deaths of 21,888 patients with imported *P. falciparum* malaria (*2*). Characteristics independently associated with death were older age, being a native of an area where malaria is nonendemic, infection occurring in eastern Africa, and absence of appropriate chemoprophylaxis. This database, including the characteristics of 832 case-patients with severe disease, provides a unique opportunity to analyze the risk factors for severe imported malaria in France. We conducted a retrospective analysis of the main features of severe imported malaria cases compared with nonsevere cases that occurred during 1996–2003 in France. Increased knowledge in this area may lead to improvement in terms of prevention and patient management.

## Materials and Methods

### Data Sources

Imported malaria is not a mandatory notifiable disease in metropolitan (mainland) France. Data for this study were collected by a reporting network of 120 selected hospital laboratories and were analyzed by the French National Reference Center for Imported and Autochthonous Malaria Epidemiology (CNREPIA). Participants of the network were asked to report imported malaria cases whenever asexual forms of *P. falciparum* were seen by laboratory observations of a patient’s blood film. Data from the national medical informatics systems and from 2 exhaustive studies (National Quality Control Survey) suggested that these cases represented 50%–55% of the total number of imported *P. falciparum* malaria cases in France during the study period (*13**,**14*). A standard 57-item questionnaire, completed by clinicians and biologists for each reported case, collected basic demographic, epidemiologic, clinical, and parasitologic information (including prophylaxis and treatment).

### Data Collection

The study population consisted of all *P. falciparum–*infected patients reported to CNREPIA during 1996–2003. We used WHO criteria for the definition of severe *P. falciparum* malaria as the primary outcome (*10**,**11*). The 1990 WHO definition (*15*) was used for cases occurring before 2000 (Table 1); the revised 2000 definition (*16*) was used for those occurring after 1999 (Table 2).

**Table 1:** Criteria used before 2000 to define severe malaria, 1990 World Health Organization definition

Major criteria
Unrousable coma
Glasgow Coma Scale score of <9
Repeated generalized seizures
Circulatory collapse, systolic blood pressure <80 mmHg despite adequate volume repletion
Pulmonary edema with presence of criteria for acute respiratory distress syndrome or acute lung injury
Spontaneous bleeding and/or disseminated intravascular coagulation
Acidemia, pH <7.35, or acidosis, serum bicarbonate <15 mmol/L
Severe anemia, hemoglobin <5 g/dL
Renal impairment, serum creatinine >265 μmol/L
Hypoglycemia, blood glucose, <2.2 mmol/L
Macroscopic hemoglobinuria (if unequivocally related to malaria)
Minor criteria
Impaired consciousness but rousable
Extreme weakness
Temperature >40°C
Parasitemia >5%
Jaundice or total bilrubin >50 μmol/L

**Table 2:** Criteria for severe malaria, World Health Organization definition revised in 2000

Extreme weakness
Impaired consciousness, Glasgow Coma Scale score <9
Pulmonary edema with presence of criteria for acute respiratory distress syndrome or acute lung injury
Repeated generalized seizures, >2 within 24 h
Circulatory collapse
Systolic blood pressure <80 mmHg despite adequate volume repletion
Spontaneous bleeding and/or disseminated intravascular coagulation
Jaundice or total bilirubin >50 μmol/L
Macroscopic hemoglobinuria (if unequivocally related to malaria)
Severe anemia
Hemoglobin ,<5 g/dL
Hypoglycemia, blood glucose <2.2 mmol/L
Acidemia, pH <7.35, or acidosis, serum bicarbonate <15 mmol/L
Hyperlactatemia, arterial lactate >5 mmol/L
Acute renal failure, urine output of <400 mL/24 h and serum creatinine >265 μmol/L
Parasitemia, >4%

In the 1990 WHO definition, only major criteria were used to define severe malaria. In the revised WHO definition in 2000, major and minor criteria were grouped together (except temperature criteria) to expand the definition of severe malaria. In addition, 2 criteria were changed: acute renal failure with urine output of <400 mL/24 h and serum creatinine >265 μmol/L and >2 generalized seizures within 24 h.

The case severity rate per 100 patients was calculated for all relevant exposure variables. Various exposure categories created for the first study (*2*) were used in this analysis. Patients were divided into categories: European travelers (persons born and residing in a country in Europe not endemic for malaria); European expatriates (persons born in a country in Europe where malaria is not endemic and residing in a sub-Saharan African country where malaria is endemic); African travelers (persons born in a sub-Saharan African country where malaria is endemic and residing in France or another country where malaria is not endemic); African residents (persons born and residing in a sub-Saharan African country where malaria is endemic); and others. Use of malaria chemoprophylaxis was categorized as reported by patients: no use; use of inappropriate drugs (chloroquine, proguanil alone, pyrimethamine, and sulfadoxine-pyrimethamine); and use of appropriate drugs (mefloquine, atovaquone-proguanil, doxycycline, and chloroquine-proguanil) according to recommendations from the Haut Conseil de la Santé Publique (www.hcsp.fr).

### Data Analysis

Logistic regression was used to identify factors associated with severe malaria and to estimate odds ratios (ORs) and 95% confidence intervals (CIs) for the association between exposure variables and severe cases. Dummy variables were used for variables with >2 categories. Variables with p values <0.25 were introduced in the multivariate logistic regression model. A manual backward stepwise approach was used to remove nonsignificant variables; only variables with p values <0.05 were retained in the final model. Interactions were sought by introducing interaction terms in the logistic regression model and testing for their significance at the 0.05 level. Moreover, because our data ranged from 1996 through 2003 and we used 2 WHO definitions for severe disease (*15**,**16*), we systematically tested interactions between year of diagnosis and all relevant associations; all analyses were adjusted for the year of diagnosis. Statistical analysis was performed by using Stata 10 (StataCorp LP, College Station, TX, USA).

## Results

During the 1996–2003 study period, 27,085 malaria cases were reported to CNREPIA. Of these, 21,888 cases were *P. falciparum* malaria (the study population), among which 20,431 (93.3%) were uncomplicated, 832 (3.8%) were severe, 433 (2%) were asymptomatic, 160 (0.7%) showed hyperreactive malarial splenomegaly, and 32 (0.1%) were unspecified. Among the 21,888 *P. falciparum* cases, 96 deaths (case-fatality rate [CFR] of 0.4%) were related to malaria. The annual number of malaria cases reported by the network increased to ≈3,000 cases up to 1999–2000 and then incidence began to regularly decrease. The CFR of severe *P. falciparum* malaria was higher during 2000–2003 than before, reaching 15.3% in 2003 (Table 3).

**Table 3:** Distribution of *Plasmodium falciparum* malaria cases and severe cases by calendar year, France, 1996–2003*

Year	*P. falciparum* malaria cases	No. (%) severe cases	Deaths	CFR for all cases, %	CFR for severe cases, %
1996	1,804	96 (5.3)	8	0.4	8.3
1997	2,057	94 (4.5)	10	0.5	11.0
1998	2,459	115 (4.7)	9	0.3	7.8
1999	3,385	118 (3.5)	9	0.2	7.6
2000†	3,355	84 (2.5)	12	0.3	14.3
2001	3,035	90 (3.0)	13	0.4	14.4
2002	2,919	105 (3.6)	15	0.5	14.3
2003	2,874	130 (4.5)	20	0.7	15.3
Total	21,888	832 (3.8)	96	0.4	11.3

*CFR, case-fatality rate. †Year World Health Organization revised definition for *severe* *P. falciparum* malaria.

Most patients were male (sex ratio M:F = 1.7), and the median age was 29.6 (range 0–96) years. African travelers were most numerous (44.6%), followed by European travelers (26.5%), African residents (12.9%), and European expatriates living in Africa (5.4%); “others” represented 10.6%. Most patients (97.5%) became infected with malaria in Africa: 59.2% in western Africa, 26.2% in central Africa, 11.2% in Madagascar and the Comoros Islands, and 0.9% in eastern Africa (Table 4; Table A1). Others (2.5%) had returned mainly from French Guiana, Haiti, India, Sri Lanka, Thailand, and Indonesia. Almost one third of the patients (30.4%) stated they had taken an appropriate chemoprophylaxis, whereas more than half stated they had not. The median duration of stay was 32 days (interquartile range [IQR] 21–62 days). The median time from return to symptom onset was 6 days (IQR 1–12 days), and 11% of patients had their first symptom before returning to France. The median time from symptom to diagnosis was 3 days (IQR 1–6 days). Most patients went to a hospital first (69.4%) and, compared with Europeans, Africans were more likely to do so (73% vs. 62.7% p<0.001). At the time of diagnosis, 7.9% of patients had high-level parasitemia (>5% of parasitized erythrocytes). Seventy-five percent of patients were hospitalized, and the median hospitalization length was 3 days (range 0–169 days). Laboratory results for 13.1% of patients revealed low platelet counts (<50 × 10^9^ cells/L); 6% of patients had low hemoglobin levels (<8 g/dL).

**Table 4:** Distribution of imported *Plasmodium falciparum* malaria cases by country of acquisition, France, 1996–2003

Country	P. falciparum malaria cases	No. (%) severe cases
Comoros	2,017	28 (1.4)
Cameroon	2,707	76 (2.8)
Congo	885	25 (2.8)
Guinea	823	24 (2.9)
Central African Republic	728	25 (3.4)
Côte d’Ivoire	4,623	160 (3.5)
Togo	604	21 (3.5)
Ghana	194	7 (3.6)
Benin	1,012	39 (3.8)
Mali	2,124	83 (3.9)
Gabon	671	32 (4.8)
Senegal	2,234	108 (4.8)
Mauritania	96	5 (5.2)
Burkina Faso	740	41 (5.5)
Madagascar	432	34 (7.8)
Niger	152	12 (7.9)
Tanzania	38	4 (10.5)
Guinea-Bissau	50	6 (12)
Nigeria	123	15 (12.2)
Mozambique	29	4 (13.8)
Kenya	101	16 (15.8)
Equatorial Guinea	31	5 16.1)
Djibouti	12	2 (16.7)
Cape Verde	4	1 (25)
Other	1,458	59 (4.1)
Total	21,888	832 (3.8)

### Description of Severe Cases

A total of 832 patients had severe malaria according to WHO definition (51% recorded before the revision in 2000). From 1996 through 2003, the evolution of the number of severe cases was the inverse of that of the total number of imported *P. falciparum* cases. Since 2000, we observed a slow decrease in the total number of imported *P. falciparum* cases, whereas severe cases increased in number and proportion (Table 3).

Of the 832 patients who had severe malaria, 386 (46.4%) were European travelers, 98 (11.8%) European expatriates, 190 (22.8%) African travelers, 73 (8.8%) African residents, and 85 (10.2%) others. Sex ratio (M:F) was 2.3, and the median age was 38.2 years (range 0–92 years). Twenty-seven patients (3.2%) were >70 years of age, and 127 (15.3%) were <15 years of age. With a 15.3% CFR, eastern Africa accounted for the highest proportion of severe cases. Two-hundred-fourteen (27.9%) of these patients stated they had taken an appropriate chemoprophylaxis. Initial visit to a general practitioner instead of a hospital occurred for 351 (43.8%) patients. A total of 803 patients (98.5%) were hospitalized, 540 (66.2%) in an intensive care unit; the median hospitalization length was 6 days (range 0–169 days). The description of the 96 fatal cases has been reported elsewhere (*2*).

### Factors Associated with Severe Cases

The CFR was 3.8% during the study period and changed over time (decrease from 1996 to 2000, then an increase since; p<0.0001 by standard χ^2^ test or trends test). Many factors were associated with an increased risk of severe malaria in univariate analysis, including older age, male gender, European origin, infection in East Africa, short stays (<21 days), inappropriate chemoprophylaxis, initial visit to a general practitioner, time to diagnosis, and diagnosis during the fall–winter season (Table A1).

However, short stays and male gender were no longer predictive of severity after controlling for all the variables, including year of diagnosis. Table 5 shows the results of multivariate analysis. Independent factors associated with severe malaria were no different whether patients were African or European, and the year of diagnosis did not modify the effect of the associations.

**Table 5:** Factors independently associated with severe malaria among patients treated for *Plasmodium falciparum* malaria in hospitals, France, 1996–2003*

Variables	Odds ratio (95% confidence interval)	p value
Age group, y		
<15	1	<0.0001
16–30	0.9 (0.7–1.2)	
31–45	1.06 (0.8–1.3)	
46–60	1.8 (1.4–2.3)	
>60	2.7 (2.0–3.6)	
Origin and residence		
African travelers	1	<0.0001
African residents	1.5 (1.1–1.9)	
European travelers	3.2 (2.6–3.8)	
European expatriates	3.7 (2.9–4.9)	
Others	1.9 (1.5–2.6)	
Region of malaria acquisition		
Western Africa	1	<0.0001
Central Africa	0.8 (0.7–0.9)	
Eastern Africa	2.6 (1.7–4.1)	
Austral Africa	1.1 (0.6–2.2)	
Madagascar and Comoros Islands	0.7 (0.5–0.9)	
Others	0.9 (0.6–1.5)	
Chemoprophylaxis		
Appropriate drugs†	1	0.001
No chemoprophylaxis	1.3 (1.1–1.5)	
Inappropriate drugs‡	1.5 (1.2–1.9)	
Place of first visit		
Hospital	1	
General practitioner	1.4 (1.2–1.7)	<0.0001
Time between onset and diagnosis, d		
<1	1	<0.0001
2–3	0.9 (0.8–1.2)	
4–6	1.6 (1.3–1.9)	
7–12	1.5 (1.1–1.8)	
>12	0.7 (0.5–0.9)	
Symptom onset		
After return to France	1	
Before return to France	1.2 (1.01–1.5)	0.03
Season of diagnosis		
Spring–summer	1	
Fall–winter	1.3 (1.2–1.5)	<0.0001

*N = 21,888. †Appropriate chemoprophylactic drugs were mefloquine, atovaquone-proguanil, doxycycline, and chloroquine-proguanil. ‡According to national recommendations, inappropriate chemoprophylactic drugs were chloroquine, proguanil, pyrimethamine, and sulfadoxine-pyrimethamine.

## Discussion

With a mean of >4,000 cases per year during the study period, France is the country reporting the highest number of imported malaria cases; >80% of cases are caused by *P. falciparum*, the species responsible for almost all severe cases and death in travelers. African travelers are particularly at risk of acquiring malaria when visiting friends and relatives. Together with African residents who declare malaria during a stay in France, African travelers accounted for 57.5% of imported malaria cases in our series. These populations, either immune or possessing residual immunity, are less likely to acquire severe cases of malaria (*9*), which may explain why the total proportion of severe cases in our series, fluctuating according to years from 2.5% to 5.3% (mean 3.8%), appears lower than those reported in previous studies (*3**–**5**,**7**,**10**–**12*).

We identified 7 risk factors independently associated with severe imported *P. falciparum* malaria; 4 were the same as those associated with fatal imported malaria (older age, being a native from an area where malaria is nonendemic, infection occurring in East Africa, and absence of appropriate chemoprophylaxis), and 3 were new risk factors (first visit was to a general practitioner, time to diagnosis 4–12 days, and diagnosis during the fall–winter season). Increasing age is a well-recognized risk factor for severe (*3**–**6**,**17*) and fatal *P. falciparum* malaria (*2**,**4**,**14**,**18**,**19*). For persons >45 years of age, age appeared more as an increased risk factor rather than a gradual increase over the entire age spectrum. This finding is contrary to what has already been reported for death, or for severe cases in another study, which included only European patients (*2**,**3*). Severe malaria was particularly frequent among nonimmigrants, as previously reported in a smaller series (*7**–**9**,**12*). These results are consistent with the hypothesis of persistent acquired immunity, even after several years of nonexposure (*12*), which may partly protect African travelers from severe malaria (*9**,**12*).

Genetic factors may also partly explain the relative protection of African travelers compared with Europeans, as noted by Lewis et al. (*8*). Five countries, Côte d’Ivoire, Cameroon, Senegal, Mali, and the Comoros Islands, accounted for the largest numbers of malaria cases; case severity rates varied between 1.4% and 4.8%. Travelers returning from the Comoros Islands were almost exclusively migrants who were visiting friends and relatives, which may explain the particularly low proportion of severe cases observed for this country. Conversely, countries in eastern Africa such as Djibouti, Kenya, Mozambique, and Tanzania accounted for fewer cases but displayed a disproportionately high number of severe cases (10.6%–16.7%). This observation confirmed what has been reported for CFR (*2*).

As suggested by a preliminary study (*17*), a relationship between *P. falciparum* drug-resistance level and severity of imported cases may exist. Because countries in eastern Africa usually harbor high proportions of drug-resistant *P. falciparum* strains (*20**,**21*), further specific studies are required to assess this point. The risk of severe malaria was higher when antimalarial chemoprophylaxis was absent or inappropriate. These results are in agreement with previous observations (*5**,**6**,**8*) and advocate strengthening the message of prevention through the use of antimalarial chemoprophylaxis. Even when this prophylaxis fails to prevent malaria because of lack of observance, drug resistance, or pharmacologic hazards, antimalarial chemoprophylaxis may confer a degree of protection against the severe form of malaria.

Management of patients who had uncomplicated malaria was not standardized among the different hospitals of our network. Depending on local procedures or on individual evaluations, patients were hospitalized, usually for the duration of their treatment, or were treated on an outpatient basis. Nevertheless, each patient with severe malaria needed to be hospitalized in an intensive care unit (exceptions to this rule depend on local medical practice). The odds of severe malaria developing were increased by 40% when the patient’s initial visit was to a general practitioner rather than to a hospital. This effect remained after controlling for time to diagnosis and suggests that this association was not due to a simple delay of diagnosis.

Time between onset of symptoms and diagnosis of 4–12 days was associated with an increased risk of severe malaria. Shorter delays of diagnosis enabled prompt treatment of malaria episodes and probably prevented their potential evolution towards severity. Diagnosis >12 days postsymptom onset usually reflected controlled parasitemia and uncomplicated malaria. These data emphasized once again the need for early diagnosis and prompt therapy. The higher severity found during the fall–winter season has been related previously to a potential mismanagement of malaria patients initially misdiagnosed with influenza (*22**,**23*). However, delay in diagnosis does not totally explain higher severity because the association between seasons and severity was not reduced after time to diagnosis was controlled for in multivariate analysis. Moreover, the influenza season in France is shorter than the fall–winter season considered in our analysis. Multivariate analysis in another study (E. Seringe et al., unpub. data) showed that malaria episodes around the end of the transmission season in areas where malaria is endemic were significantly associated with an increased risk of death (OR for each additional month away from the end of the malaria season 0.75, 95% CI 0.64–0.87; p<0.001). If one considers the geographic origin of the largest number of imported malaria cases, the end of the transmission season matched the French winter season. An additional factor, symptom onset before return to France, was marginally independently associated with severe malaria (OR 1.20, 95% CI 1.01–1.50; p = 0.03). Repatriations for medical reasons, including malaria infections, may partially explain this result.

This study has several limitations. Our surveillance network accounted for only 50%–55% of total malaria cases (uncomplicated, severe, or fatal) imported to France. Two annual exhaustive studies (National Quality Control Survey [*24*]) and F. Legros (unpub. data) suggest that our corresponding sites had a correct representation of patients. Thus, it is unlikely that factors associated with severity in imported malaria would be different among cases not seen in our network. From 2000 through 2003, the CFR in patients infected with *P. falciparum* and the percentage of severe cases have increased from 0.3% to 0.7% (p<0.0001) and from 2.5% to 4.5% (p = 0.3) respectively, whereas the CFR of severe cases remained stable (from 14.3% to 15.3%). These figures may be caused by fluctuations over time but may also be partially explained by the revision of WHO criteria for severe malaria in 2000. This revision may have led to a better categorization of severe cases, judging by the increased odds of death associated with severity after the 2000 reclassification (OR 1.84, 95% CI 1.20–2.90). However, no interaction was identified between year of diagnosis and relevant associations and all analyses were adjusted for the year of diagnosis.

Biological data were not introduced in multivariate analysis, first, because of a large number of missing data (59% and 60% of missing data in severe cases for platelet counts and hemoglobinemia, respectively) and, second, because parasitemia (10% of missing data), hemoglobin, and platelet counts are directly or indirectly part of the definition of severe malaria. Thus, parasitemia, although strongly associated with severe forms of malaria in univariate analysis, was not introduced in the final multivariate model because it is in the causal pathway between several factors and disease. For instance, delay to diagnosis leads to high parasitemia, which itself leads to severe forms of malaria. Adjusting for high parasitemia in the final model would make the relationship between delay to diagnosis and severe malaria disappear.

Treatment of severe malaria may have varied according to the physicians. Detailed French guidelines for the management of severe *P. falciparum* malaria were available (*25*). These guidelines strongly recommended a quinine-loading dose but did not recommend exchange transfusion. Intravenous artesunate was not used in France during the study period.

The clinical course of *P. falciparum* malaria is unpredictable and may result in severe illness and death. Although the acquisition of *P. falciparum* malaria among travelers to countries where malaria is endemic can never be completely avoided, our data suggest that severe malaria may largely be prevented. Pretravel health advice promoting the compliant use of antimalaria chemoprophylaxis for every traveler, with a particular focus on nonimmune travelers and elderly persons, is essential. In addition, increased vigilance of travelers in reporting symptoms and of physicians in providing prompt diagnosis and treatment is required to reduce any delay in patient management.

## Appendix

**Table A1:** Factors associated with severe malaria among patients treated for *Plasmodium falciparum* malaria in hospitals, France, 1996–2003*

Factor	No. (%) severe cases	No. (%) patients	Odds ratio (95% confidence interval)	p value
Total	832 (3.8)	21,888	NA	NA
No. of deaths	96	NA	NA	NA
Age group, y				
<15	127 (2.5)	5,098 (23.3)	1	<0.0001
16–30	177 (2.9)	6,107 (27.9)	1.2 (0.9–1.5)	
31–45	207 (3.2)	6,421 (29.3)	1.3 (1.04–1.6)	
46–60	229 (6.7)	3,429 (15.7)	2.8 (2.2–3.5)	
>60	92 (11.04)	833 (3.8)	4.8 (3.7–6.4)	
Sex				
Female	251 (3.1)	8,091 (37)	1	
Male	579 (4.2)	13,772 (63)	1.2 (1.1–1.3)	<0.0001
Unknown	2	25	NA	NA
Origin and residence				
African travelers	190 (1.9)	9,764 (44.6)	1	<0.0001
African residents	73 (2.6)	2,814 (12.9)	1.34 (1.02–1.80)	
European travelers	386 (6.7)	5,805 (26.5)	3.60 (3.01–4.30)	
European expatriates	98 (8.3)	1,188 (5.4)	4.5 (3.5–5.8)	
Others	85 (3.7)	2,317 (10.6)	1.9 (1.5–2.5)	
Region of malaria acquisition				
Western Africa	527 (4.1)	12,941 (59.1)	1	<0.0001
Central Africa	182 (3.2)	5,738 (26.2)	0.8 (0.6–0.9)	
Eastern Africa	27 (15.3)	176 (0.8)	4.3 (2.8–6.5)	
Austral Africa	10 (6.1)	165 (0.8)	1.5 (0.8–2.9)	
Madagascar and Comoros Islands	62(2.5)	2,449 (11.2)	0.6 (0.5–0.8)	
Others	22 (5.4)	410 (1.9)	1.3 (0.9–2.1)	
Unknown	2	9	NA	
Duration of stay, d				
0–21	220 (5.9)	3,748 (26.1)	1.5 (1.2–1.8)	<0.0001
22–32	115 (3.4)	3,433 (23.9)	0.8 (0.6–1.1)	
33–62	110 (2.9)	3,755 (26.1)	0.7 (0.5–0.9)	
>62	138 (4.03)	3,424 (23.8)	1	
Unknown	249 (3.3)	7,528	NA	
Chemoprophylaxis				
Appropriate drugs†	214 (3.6)	5,935 (30.4)	0.9 (0.7–1.1)	0.2
No chemoprophylaxis	403 (4)	10,097 (51.7)	1	
Inappropriate drugs‡	150 (4.3)	3,490 (17.9)	1.1 (0.9–1.3)	
Unknown	65	2,366	NA	
Season of diagnosis				
Spring–summer	448 (3.1)	14,327 (65.6.)	1	NA
Fall–winter	379 (5.1)	7,511 (34.4)	1.5 (1.3–1.7)	<0.0001
Unknown	5	50	NA	NA
Time lapse between return and onset, d				
–25 to 0	194 (4.2)	4,569 (23.1)	1	0.15
1–7	252 (3.7)	6,863 (34.8)	0.90 (0.70–1.04)	
7–14	172 (3.9)	4,415 (22.4)	0.9 (0.7–1.1)	
14–30	96 (4)	2,397 (12.1)	0.9 (0.7–1.2)	
>30	43 (2.9)	1,490 (7.5)	0.6 (0.5–0.9)	
Unknown	75 (3.5)	2,154	NA	
Time between onset and diagnosis, d				
<3	367 (3.1)	11,899 (58.4)	1	<0.0001
4–6	234 (5.7)	4,082 (20.1)	1.9 (1.6–2.3)	
7–12	125 (5.2)	2,401 (11.8)	1.7 (1.4–2.1)	
>12	57 (2.9)	1,974 (9.7)	0.9 (0.7–1.2)	
Unknown	49	1,532	NA	
Time between return and diagnosis, d				
0–5	221 (3.8)	5,838 (27.8)	1	0.09
6–10	172 (3.6)	4,851 (23.1)	0.9 (0.8–1.1)	
11–15	179 (4.4)	4,061 (19.4)	1.2 (0.9–1.4)	
>15	217 (3.5)	6,210 (29.6)	0.9 (0.8–1.1)	
Unknown	43	928	NA	
Place of first medical visit				
Hospital	450 (3.1)	14,574 (69.5)	1	NA
General practitioner	351 (5.5)	6,420 (30.5)	1.8 (1.5–2.1)	<0.0001
Unknown	31	894	NA	NA
Parasitemia, %				
<1	156 (1.3)	12,322 (67.9)	1	<0.0001
1.1–2	69 (3.2)	2,146 (11.8)	2.6 (1.9–3.4)	
2.1–5	119 (5.3)	2,249 (12.4)	4.4 (3.4–5.6)	
5.1–10	167 (19)	880 (4.8)	18.30 (14.50–23.01)	
>10.1	238 (43.6)	546 (3)	60.3 (47.8–75.9)	
Unknown	83	3,745	NA	
Hemoglobin, g/dL				
>12	125 (2.7)	4,616 (49.6)	1	<0.0001
10.1–12	76 (2.8)	2,727 (29.3)	1.02 (0.70–1.40)	
8.1–10	64 (4.6)	1,377 (14.8)	1.7 (1.3–2.4)	
<8	65 (10.9)	591 (6.3)	4.4 (3.2–6.1)	
Unknown	502	12,577	NA	
Platelet count, × 10^9^/L				
>50	132 (1.6)	8,176 (86.9)	1	<0.0001
26–50	88 (9.9)	889 (9.5)	6.7 (5.1–8.8)	
11–25	96 (32.6)	294 (3.1)	29.6 (21.9–39.8)	
<10	24 (53.3)	45 (0.5)	69.6 (37.8–128.2)	
Unknown	492	12,484	NA	
Leukocyte count, × 10^9^ cells/L				
6–10	250 (2.9)	8,418 (92.4)	1	<0.0001
10.1–15	37 (6.7)	550 (6.1)	2.3 (1.6–3.4)	
>15	25 (17.7)	141 (1.5)	7.1 (4.5–11.1)	
Unknown	520	12,779	NA	

*N = 21,888. NA, not applicable. †Appropriate chemoprophylactic drugs were mefloquine, atovaquone-proguanil,doxycycline, and chloroquine-proguanil. ‡According to national recommendations, inappropriate chemoprophylactic drugs were chloroquine, proguanil, pyrimethamine, and sulfadoxine- pyrimethamine.

## References

[1] Ryan ET, Wilson ME, Kain KC. Illness after international travel. N Engl J Med. 2002;347:505–16. 10.1056/NEJMra02011812181406

[2] Legros F, Bouchaud O, Ancelle T, Arnaud A, Cojean S, Le Bras J, Risk factors for imported fatal *Plasmodium falciparum* malaria, France, 1996–2003. Emerg Infect Dis. 2007;13:883–8.1755322810.3201/eid1306.060955

[3] Mühlberger N, Jelinek T, Behrens RH, Gjorup I, Coulaud JP, Clerinx J, Age as a risk factor for severe manifestations and fatal outcome of falciparum malaria in European patients: observations from TropNetEurop and SIMPID Surveillance Data. Clin Infect Dis. 2003;36:990–5. 10.1086/37422412684911

[4] Schwartz E, Sadetzki S, Murad H, Raveh D. Age as a risk factor for severe *Plasmodium falciparum* malaria in nonimmune patients. Clin Infect Dis. 2001;33:1774–7. 10.1086/32252211641827

[5] Calleri G, Lipani F, Macor A, Belloro S, Riva G, Caramello P. Severe and complicated falciparum malaria in Italian travelers. J Travel Med. 1998;5:39–41. 10.1111/j.1708-8305.1998.tb00456.x9772316

[6] Jensenius M, Ronning EJ, Blystad H, Bjorneklett A, Hellum KB, Bucher A, Low frequency of complications in imported falciparum malaria: a review of 222 cases in south-eastern Norway. Scand J Infect Dis. 1999;31:73–8. 10.1080/0036554995016192510381222

[7] Matteelli A, Colombini P, Gulletta M, Castelli F, Carosi G. Epidemiological features and case management practices of imported malaria in northern Italy 1991–1995. Trop Med Int Health. 1999;4:653–7. 10.1046/j.1365-3156.1999.00468.x10583898

[8] Lewis SJ, Davidson RN, Ross EJ, Hall AP. Severity of imported falciparum malaria: effect of taking antimalarial prophylaxis. BMJ. 1992;305:741–3. 10.1136/bmj.305.6856.7411422328PMC1883441

[9] Bouchaud O, Cot M, Kony S, Durand R, Schiemann R, Ralaimazava P, Do African immigrants living in France have long-term malarial immunity? Am J Trop Med Hyg. 2005;72:21–5.15728861

[10] Kain KC, Harrington MA, Tennyson S, Keystone JS. Imported malaria: prospective analysis of problems in diagnosis and management. Clin Infect Dis. 1998;27:142–9. 10.1086/5146169675468

[11] Ben-Ami R, Siegman-Igra Y, Anis E, Brook GJ, Pitlik S, Dan M, Malaria in travelers returning from short organized tours to holiday resorts in Mombassa, Kenya. Isr Med Assoc J. 2005;7:364–7.15984377

[12] Phillips A, Bassett P, Zeki S, Newman S, Pasvol G. Risk factors for severe disease in adults with falciparum malaria. Clin Infect Dis. 2009;48:871–8. 10.1086/59725819243243

[13] Legros F, Danis M. Surveillance of malaria in European Union countries. Euro Surveill. 1998;3:45–7.1263176810.2807/esm.03.05.00103-en

[14] Legros F, Pichard E, Danis M. Formes graves du paludisme d’importation: données épidémiologiques en France 1999–2001. Med Mal Infect. 2003;33(suppl B):3–5.

[15] World Health Organization, Division of Control of Tropical Diseases. Severe and complicated malaria. Trans R Soc Trop Med Hyg. 1990;84(Suppl 2):1–65. 10.1016/0035-9203(90)90363-J2219249

[16] World Health Organization. Communicable Diseases Cluster. Severe falciparum malaria. Trans R Soc Trop Med Hyg. 2000;94(Suppl 1):S1–90.11103309

[17] Belmonte O, Saliba G, Kamouh W, Fontanet A, Le Bras J. Predictive factors of severe disease secondary to falciparum malaria among travelers. Pathol Biol (Paris). 2010;1:18–24. 10.1016/j.patbio.2009.07.02120303672

[18] Krause G, Schoneberg I, Altmann D, Stark K. Chemoprophylaxis and malaria death rates. Emerg Infect Dis. 2006;12:447–51.1670478310.3201/eid1203.050736PMC3291437

[19] Greenberg AE, Lobel HO. Mortality from *Plasmodium falciparum* malaria in travelers from the United States, 1959 to 1987. Ann Intern Med. 1990;113:326–7.219791510.7326/0003-4819-113-4-326

[20] Wongsrichanalai C, Pickard AL, Wernsdorfer WH, Meshnick SR. Epidemiology of drug-resistant malaria. Lancet Infect Dis. 2002;2:209–18. 10.1016/S1473-3099(02)00239-611937421

[21] Sridaran S, McClintock SK, Syphard LM, Herman KM, Barnwell JW, Udhayakumar V. Anti-folate drug resistance in Africa: meta-analysis of reported dihydrofolate reductase (*dhfr*) and dihydropteroate synthase (*dhps*) mutant genotype frequencies in African *Plasmodium falciparum* parasite populations. Malar J. 2010;9:247. 10.1186/1475-2875-9-24720799995PMC2940896

[22] Christen D, Steffen R, Schlagenhauf P. Deaths caused by malaria in Switzerland 1988–2002. Am J Trop Med Hyg. 2006;75:1188–94.17172391

[23] Winters RA, Murray HW. Malaria—the mime revisited: fifteen more years of experience at a New York City teaching hospital. Am J Med. 1992;93:243–6. 10.1016/0002-9343(92)90228-41524074

[24] Legros F, Fromage M, Ancelle T, Burg E, Janot C, Maisonneuve P, Enquête nationale de recensement des cas de paludisme d'importation en France métropolitaine pour l'année 1997. Bull Epidemio Hebdo (France). 1999;11:41–2.

[25] Conférence de Consensus en Thérapeutique Anti-infectieuse de la Société de Pathologie Infectieuse de Langue Française (SPILF). Prise en charge et prévention du paludisme d’importation à *Plasmodium falciparum.* Med Mal Infect. 1999;29:S115–41.

# Research article: Severe Imported *Plasmodium falciparum* Malaria, France, 1996–2003

**DOI:** 10.3201/eid1705.101527

**Authors:** Elise Seringe, Marc Thellier, Arnaud Fontanet, Fabrice Legros, Olivier Bouchaud, Thierry Ancelle, Eric Kendjo, Sandrine Houze, Jacques Le Bras, Martin Danis, Rémy Durand, for the French National Reference Center for Imported Malaria Study Group

**Affiliations:** Author affiliations: University Pierre et Marie Curie, Paris, France (E. Seringe, M. Thellier, M. Danis);; Groupe Hospitalier Pitié-Salpĕtrière, Paris (E. Seringe, M. Thellier, M. Danis);; Centre National de Référence du Paludisme, Paris (M. Thellier, F. Legros, E. Kendjo, S. Houze, J. Le Bras, M. Danis, R. Durand);; Institut Pasteur, Paris (E. Seringe, A. Fontanet);; Conservatoire National des Arts et Métiers, Paris (A. Fontanet);; Hôpital Avicenne, Bobigny, France (O. Bouchaud, R. Durand);; Hôpital Cochin, Paris (T. Ancelle)

## Earning CME Credit

To obtain credit, you should first read the journal article. After reading the article, you should be able to answer the following, related, multiple-choice questions. To complete the questions and earn continuing medical education (CME) credit, please go to www.medscape.org/journal/eid. Credit cannot be obtained for tests completed on paper, although you may use the worksheet below to keep a record of your answers. You must be a registered user on Medscape.org. If you are not registered on Medscape.org, please click on the New Users: Free Registration link on the left hand side of the website to register. Only one answer is correct for each question. Once you successfully answer all post-test questions you will be able to view and/or print your certificate. For questions regarding the content of this activity, contact the accredited provider, CME@medscape.net. For technical assistance, contact CME@webmd.net. American Medical Association’s Physician’s Recognition Award (AMA PRA) credits are accepted in the US as evidence of participation in CME activities. For further information on this award, please refer to http://www.ama-assn.org/ama/pub/category/2922.html. The AMA has determined that physicians not licensed in the US who participate in this CME activity are eligible for *AMA PRA Category 1 Credits*™. Through agreements that the AMA has made with agencies in some countries, AMA PRA credit is acceptable as evidence of participation in CME activities. If you are not licensed in the US and want to obtain an AMA PRA CME credit, please complete the questions online, print the certificate and present it to your national medical association.

## Article Title: Severe Imported *Plasmodium falciparum* Malaria, France, 1996–2003

## CME Questions

**1. You are seeing a 30-year-old man with fever for 5 days. He returned from a trip to Kenya 3 days ago.Which of the following statements regarding malaria among travelers is most accurate?**A. Malaria is the most common cause of fever among travelers returning from the developing worldB. Most cases of malaria among returning travelers are classified as severeC. Death due to malaria, even severe malaria, is extremely rare among returning travelersD. Adults from endemic areas are at the highest risk for death from malaria acquired during travel to areas with a high prevalence of malaria**2. You suspect malaria in the patient described in question 1. What were characteristics of patients with malaria in the current study?**A. Malaria was most common among European travelersB. Most patients had received appropriate chemoprophylaxisC. Most patients had symptoms before returning from travelD. 75% of patients were hospitalized**3. The patient in question 1 is diagnosed with malaria. Which of the following demographic and travel variables was most associated with a higher risk for severe malaria in the current study?**A. Male sexB. African originC. Travel to East AfricaD. Lower socioeconomic status**4. Which of the following medical variables was most associated with a higher risk for severe malaria in the current study?**A. ThrombocytopeniaB. Initial presentation to a hospitalC. Absence of chemoprophylaxisD. Normal serum leukocyte count

## Activity Evaluation

**Table:** 

1. The activity supported the learning objectives.				
Strongly Disagree				Strongly Agree
1	2	3	4	5
2. The material was organized clearly for learning to occur.				
Strongly Disagree				Strongly Agree
1	2	3	4	5
3. The content learned from this activity will impact my practice.				
Strongly Disagree				Strongly Agree
1	2	3	4	5
4. The activity was presented objectively and free of commercial bias.				
Strongly Disagree				Strongly Agree
1	2	3	4	5

## Supplementary Material

Technical Appendix Members of the National Reference Center Study Group for Imported Malaria in Metropolitan France.
